# Supplementary material for: Raman-guided subcellular pharmaco-metabolomics for metastatic melanoma cells
Source: Nat Commun. 2020 Sep 24;11:4830. doi: 10.1038/s41467-020-18376-x (PMC7518429; doi:10.1038/s41467-020-18376-x)
Supplement: Supplementary file 1 — Supplementary Information [file 41467_2020_18376_MOESM1_ESM.pdf]

Supplementary Information

Raman-guided Subcellular Pharmaco-Metabolomics for Metastatic Melanoma Cells

Du *et al*

Supplementary Figures 1 - 13

Supplementary Tables 1 – 2

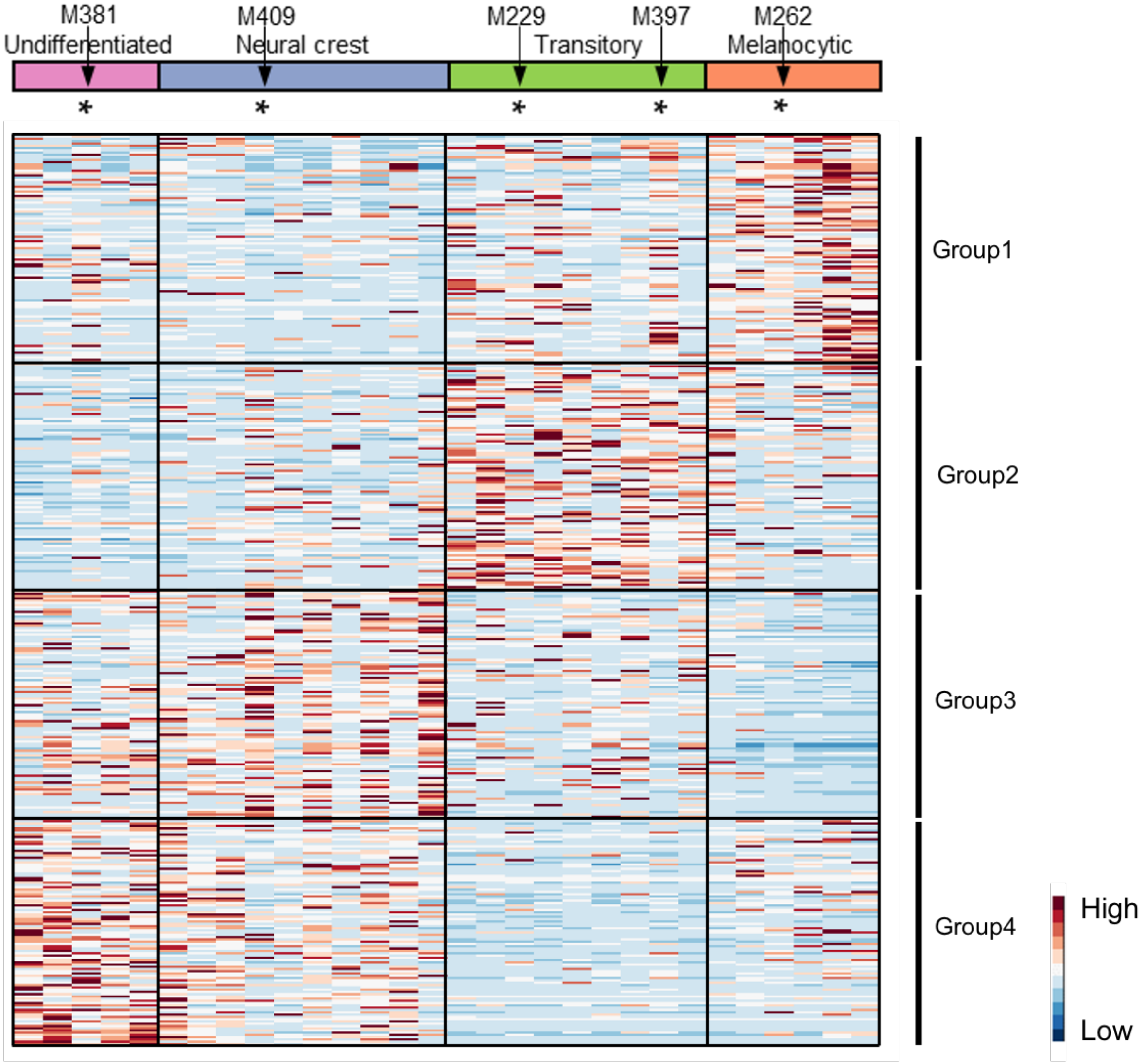

Supplementary Figure 1. Heatmap of the top 100 metabolic genes that are upregulated in each phenotype.

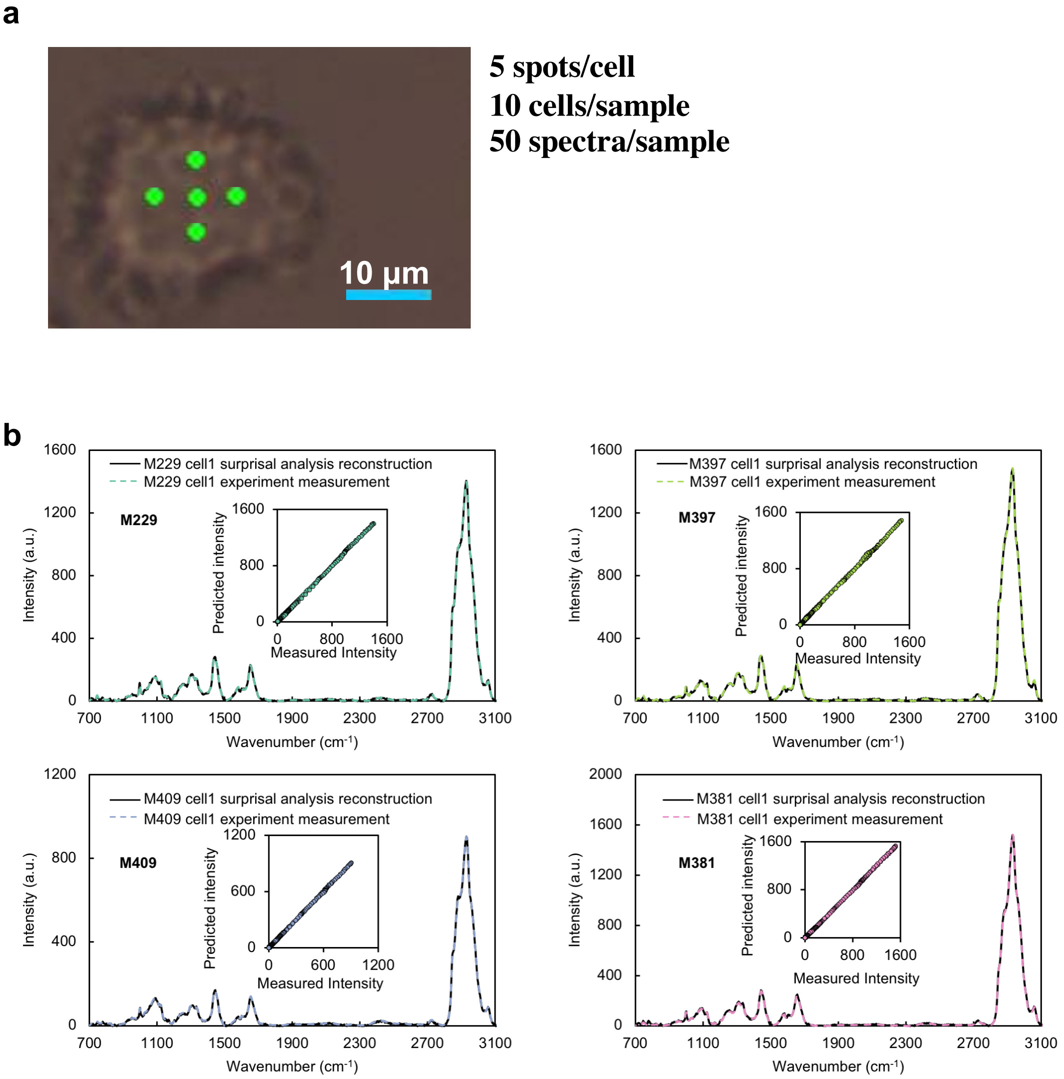

Supplementary Figure 2. Acquisition and surprisal analysis (SA) of spontaneous Raman spectra across the cell lines. a, Illustration of laser focal spots on a representative cell under widefield mode during spontaneous Raman spectra acquisition. Green points indicate the laser focal points on cells. We selected 5 points (center, top, bottom, left, right) on each cell to acquire Raman spectra and averaged them for one cell. We randomly chose 10 cells to represent one cell line. **b,** The comparison between SA reconstructed and experimentally obtained Raman spectra for individual M229, M397, M409, M381 cells. The SA plots are constructed by summing the spectral distribution and amplitudes of the first five resolved constraints (λ_0 - λ_4). The inset plots show the correlation between the predicted and calculated Raman spectra.

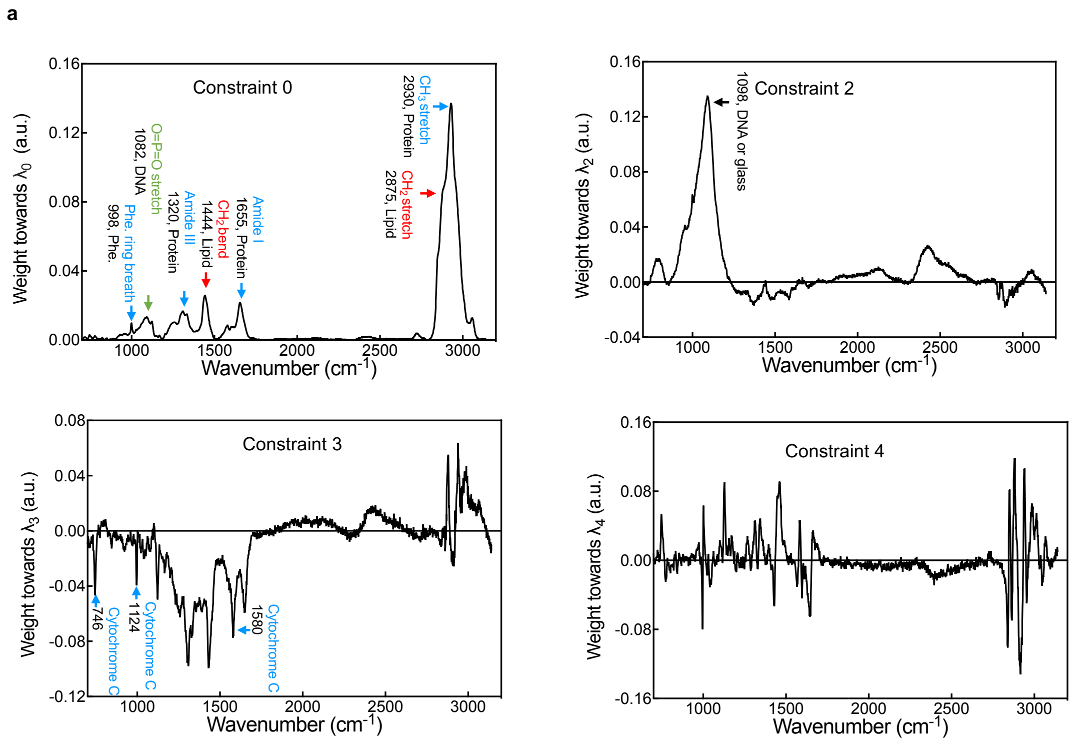

Supplementary Figure 3. Raman peak assignments of the shared constraint (constraint 0, λ_0) and lower amplitude constraints (constraints 2-4, λ_2 - λ_4).

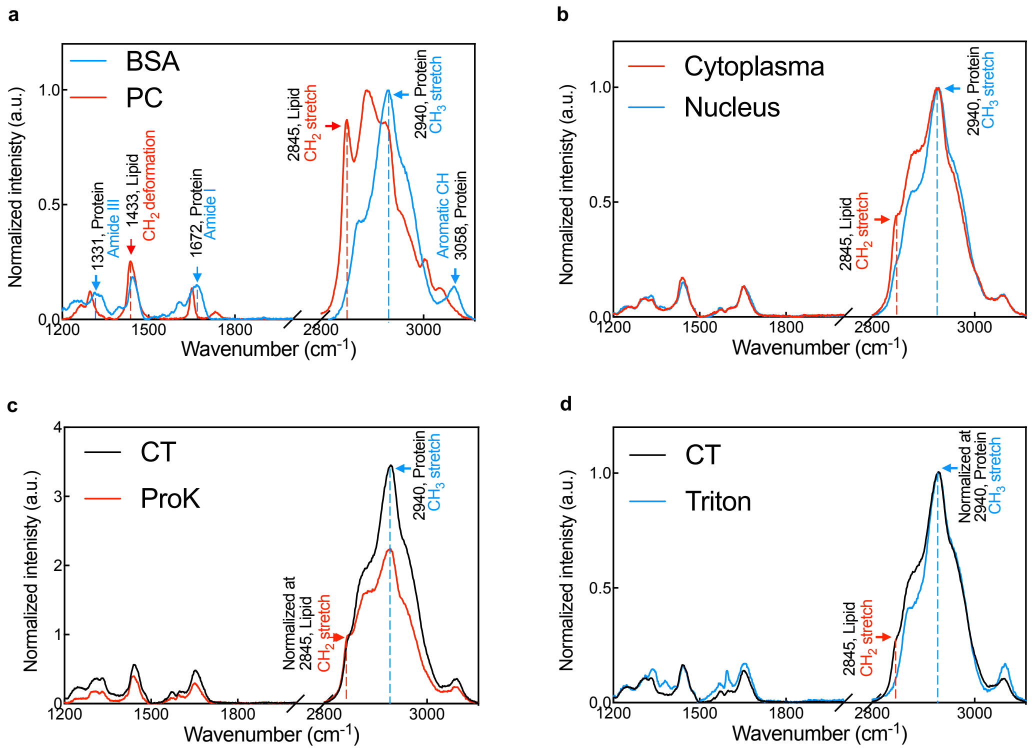

Supplementary Figure 4. Validation of the assignments for the 2845 and 2940 cm^{-1} Raman peaks to lipids and proteins in Fig. 1g. a, Raman spectra of pure proteins (bovine serum albumin, BSA, blue) and lipids (1,2-dioleoyl phosphocholine, the highest abundant lipid in M381 cells from lipidomics, PC, red). 2845 cm^{-1} and 2940 cm^{-1} peaks are dash-line highlighted (the same for all sub-figures). **b,** Raman spectra from cytoplasm (red, more lipid rich) and nucleus (blue, more protein rich) of M381 cells. **c,** Raman spectra of M381 cells before (CT, control, black) and after protease k (ProK, red) treatment to digest most of the proteins. **d,** Raman spectra of M381 cells before (CT, black) and after triton (Triton, red) treatment to wash away most lipids. Spectra in a) and b) are self-normalized, in c) and d) are normalized to 2845 cm^{-1} and 2940 cm^{-1}, respectively.

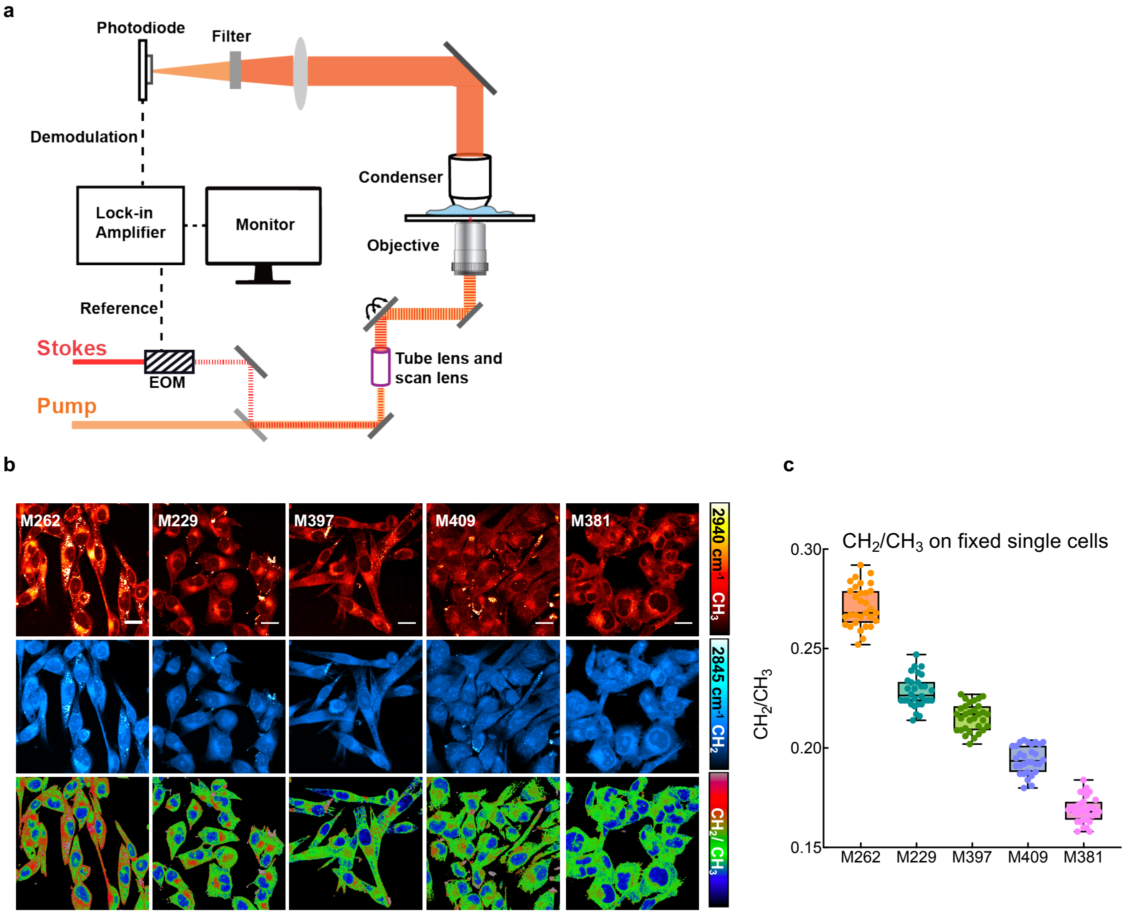

Supplementary Figure 5. SRS imaging of fixed melanoma cells. a, Illustration of SRS configuration. **b,** Representative SRS imaging of fixed melanoma cells at CH_2 (top, 2845 cm^{-1}) and CH_3 (middle, 2940 cm^{-1}) channels. Ratiometric images (bottom, CH_2/CH_3) were generated from the same sets of CH_2 and CH_3 images. Scale bar: 20 µm. **c,** Average single-cell CH_2/CH_3 values from fixed cells for 5 cell lines (n = 30 cells per cell line examined over 3 independent experiments). Data are plotted as boxplots: center line indicates median; box limits indicate upper and lower quartiles; whiskers indicate minimum and maximum. Source data are provided as a Source data file.

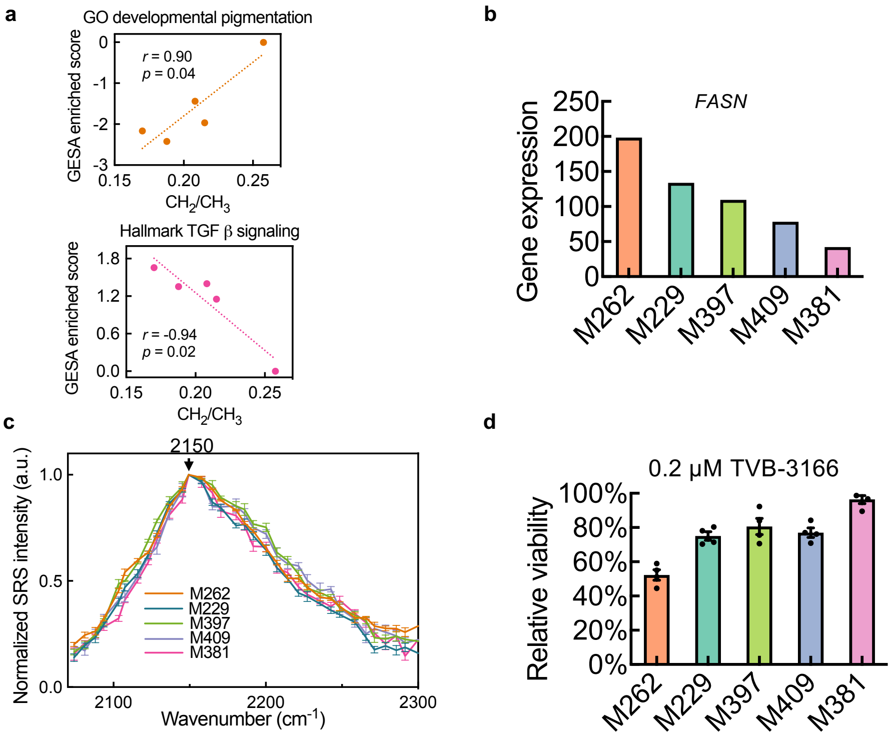

Supplementary Figure 6. The identification of *de novo* fatty acid synthesis as a druggable susceptibility for more differentiated melanoma cells. a, Top panel: cell line-dependent correlation between GSEA scores for the GO developmental pigmentation pathway and CH_2/CH_3 ratios. Bottom panel: cell line-dependent correlation between GSEA scores for TGF beta signaling pathway and CH_2/CH_3 ratios. Each dot represents a two-dimensional relationship for one cell line. **b,** Expression level of FASN across the five different melanoma cell lines (n = 1). **c,** C-D region hyperspectral SRS (hSRS) spectra on cytoplasm in each single d_7-glucose labeled melanoma cells for five cell lines (n = 6, 8, 6, 8, 9 for M262, M229, M397, M409, M381 respectively, data shown as mean ± SEM, spectra are self-normalized). d, Viability tests of melanoma cell lines under 0.2 μM TVB-3166 (FASN inhibitor) treatment for 3 days (n = 4 independent experiments, data shown as mean ± SEM). Source data are provided as a Source data file.

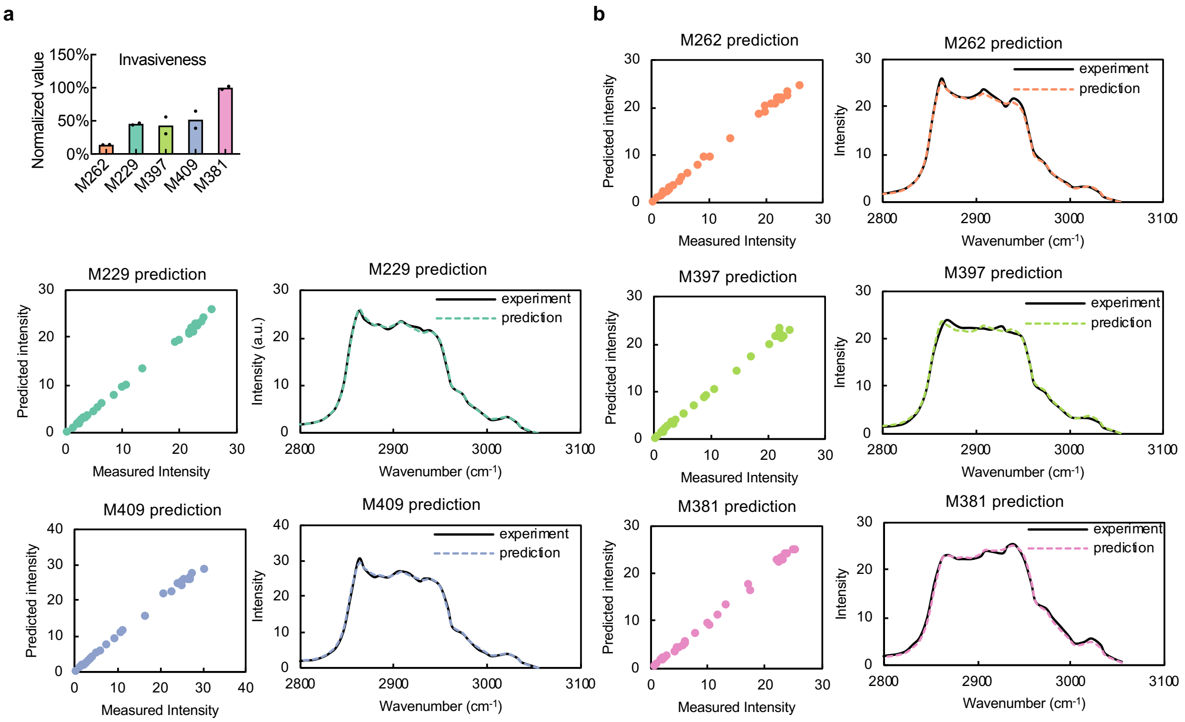

Supplementary Figure 7. Invasiveness assay and validation of SA of hSRS spectra from lipid droplets (LDs). a, Relative invasiveness of different melanoma cell lines quantified by trans-well assay (n = 2 independent experiments). **b,** The comparison between SA reconstructed and experimentally obtained Raman spectra for randomly selected individual lipid droplets from M262, M229, M397, M409, M381 cells. The SA reconstructions utilized only the first two constraints λ_0 and λ_1. Source data are provided as a Source data file.

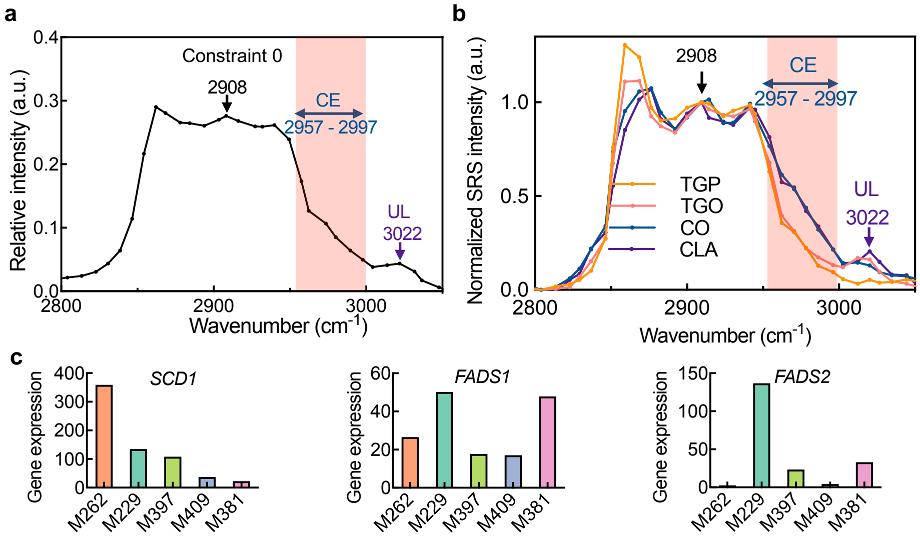

Supplementary Figure 8. Validation of peak assignments in Fig. 3e, and gene expression of fatty acid desaturases across cell lines. a, Peak assignments of constraint 0 (λ_0). The 2908 cm^{-1} reference peak, cholesteryl esters band (CE, 2957 cm^{-1} to 2997 cm^{-1}) and unsaturated lipids peaks (UL, 3022 cm^{-1}) are indicated. **b,** SRS spectra for pure references of glyceryl tripalmitate (TGP), glyceryl trioleate (TGO), cholesteryl oleate (CO), cholesteryl linoleate (CLA) after normalization at 2908 cm^{-1}. The broad band (indicated by pink shadow) ranging from 2957 cm^{-1} to 2997 cm^{-1} features CO and CLA, and is therefore assigned to cholesteryl esters. The 3022 cm^{-1} peak (indicated by violet arrow) is assigned to unsaturated lipid (=C-H), distinct in TGO, CO, CLA. **c,** Gene expression levels of desaturase SCD1, FADS1 and FADS2 across cell lines (n = 1).

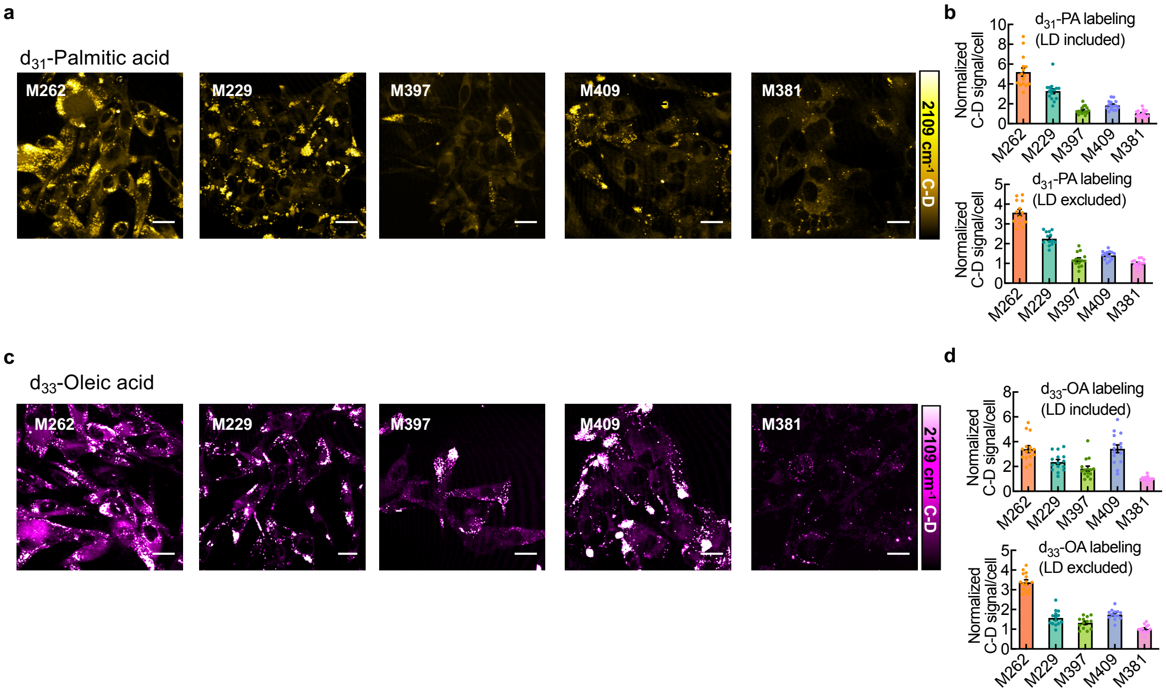

Supplementary Figure 9. Deuterated fatty acids uptake across cell lines. a, Representative C-D channel (at 2109 cm^{-1}) SRS imaging of melanoma cells by incubating cells with d_{31}-palmitic acid (50 µM, 3 days). **b,** Quantification of relative C-D signals in d_{31}-palmitic acid labeling cells at the single-cell level including or excluding LDs (n = 15 cells examined from 3 independent experiments, the C-D signal of M381 cells is normalized to 1). **c,** Representative C-D channel (at 2109 cm^{-1}) SRS imaging of melanoma cells by incubating cells with d_{33}-oleic acid (50 µM, 3 days). **d,** Quantification of relative C-D signals in d_{33}-oleic acid labeling cells at the single-cell level including or excluding LDs (n = 15 cells examined over 3 independent experiments. The C-D signal of M381 cells is normalized to 1. Scale bar, 20 µm. Data shown as mean ± SEM. Source data are provided as a Source data file.

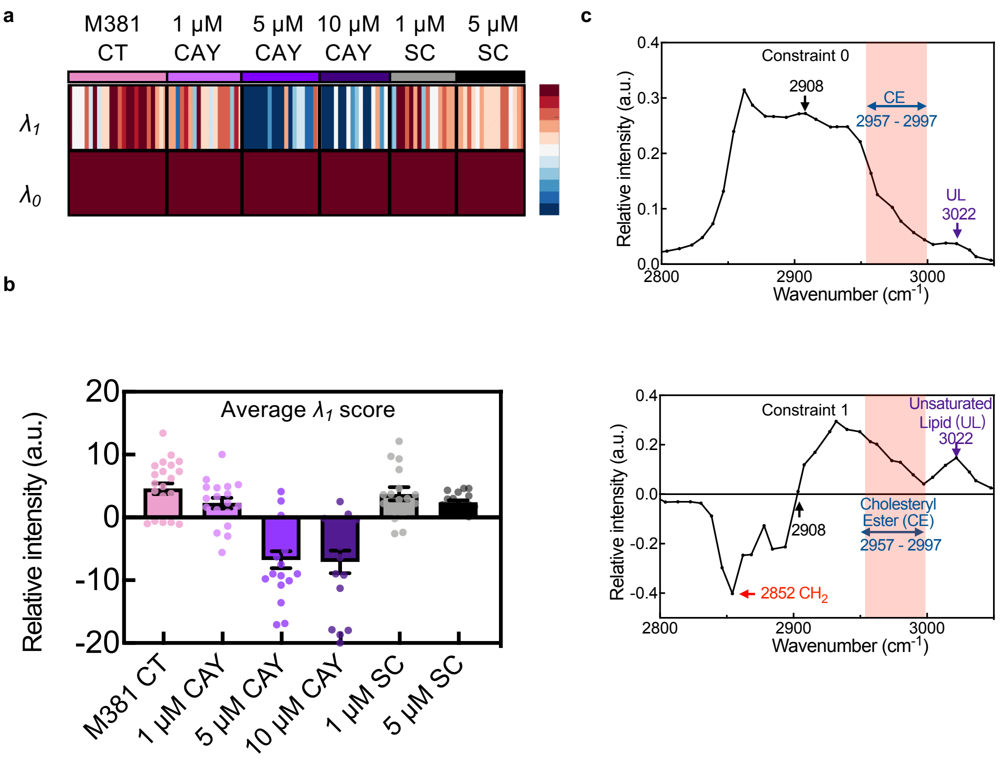

Supplementary Figure 10. SA of hSRS spectra on single LDs in M381 cells with drug treatment. a) Heatmap for scores of the top two constraints (constraint 0 (*λ0*) – constraint 1 (*λ1*)) by surprisal analysis of hSRS spectra on LDs in M381 cells at different treatment conditions, i.e. M381 control (CT), 1 μM CAY, 5 μM CAY, 10 μM CAY, 1 μM SC, 5 μM SC. Each column represents an individual LD and each row represents the constraint scores. n = 24, 18, 19, 17, 16, 16 for M381 CT, 1 μM CAY, 5 μM CAY, 10 μM CAY, 1 μM SC, 5 μM SC respectively, examined over 3 independent experiments. b) The average score of λ_1 in a) across six treatment conditions. Data shown as mean ± SEM. c) Raman peak assignments for λ_0 and λ_1. The 3022 cm^{-1} peak (violet arrow) is assigned to unsaturated lipids (UL) and the pink shadowed range from 2957 cm^{-1} to 2997 cm^{-1} is assigned to cholesteryl esters (CE). 2908 cm^{-1} is the zero point in λ_1. Source data are provided as a Source data file.

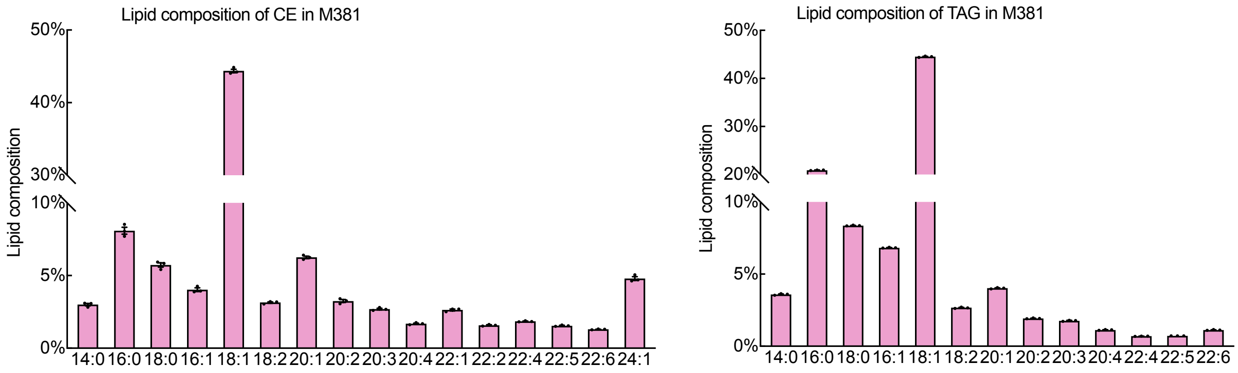

Supplementary Figure 11. Lipidomics of CE and TAG from M381 cells show the involvement of both MUFA and PUFA. Bulk lipidomics of M381 cells with relative percentages of different fatty acid chains in CE (n = 3 independent experiments, left) and in TAG (n = 3 independent experiments, right). Data shown as mean ± SEM. Lipidomics data are provided as Supplementary Data 1. Source data are provided as a Source data file.

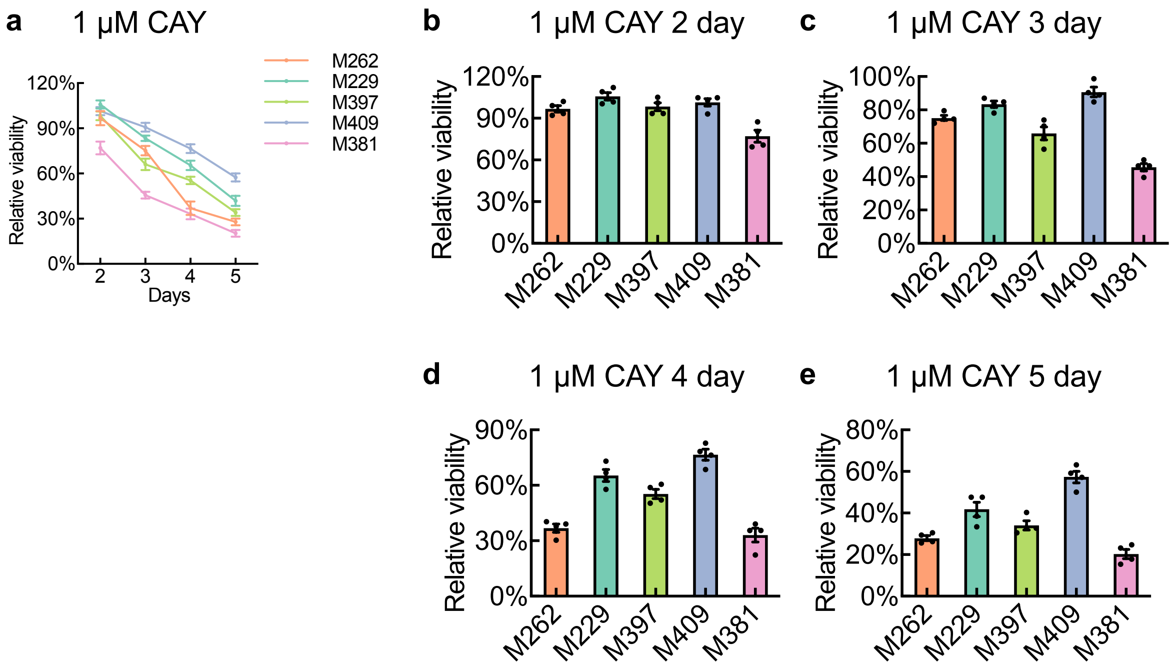

Supplementary Figure 12. Viability assays of melanoma cell lines under different days of 1 μM CAY treatment. a, Dependence of viability for cell lines on the treatment length with 1 μM CAY. **b-e,** Bar-chart plot for comparing viability in (a) across five cell lines after 2-day (b), 3-day (c), 4-day (d), and 5-day (e) of CAY treatment. n = 4 independent experiments, Data shown as mean ± SEM. Source data are provided as a Source data file.

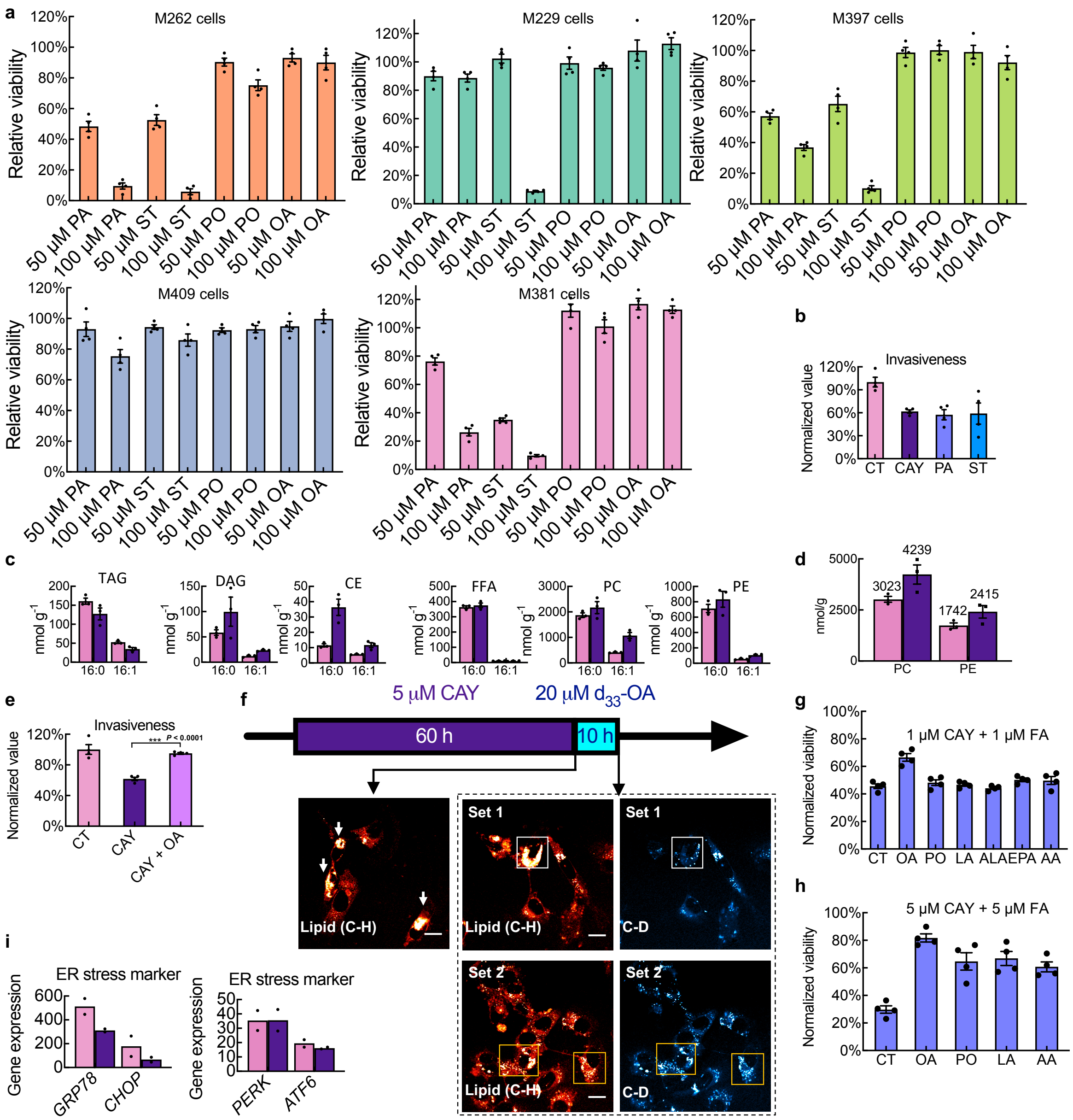

Supplementary Figure 13. The mesenchymal M381 is sensitive to saturated fatty acids (SFA) related lipotoxicity. a, Relative viability of cells treated with major SFAs (PA, 16:0 and ST, 18:0), and major MUFAs (PO, 16:1 and OA, 18:1) at indicated concentration for 3 days (n = 4 independent experiments). **b,** Relative invasiveness of control M381 cells (normalized to 100%) and M381 cells after treating with 1 μM CAY, 50 μM PA or 50 μM ST for 3 days. n = 4 independent experiments. **c,** Concentration changes of PA (16:0) and PO (16:1) fatty acid chains in the 6 main species of lipids with (3 days, CAY, purple) and without (CT, pink) CAY treatment from lipidomics (n = 3 independent experiments). **d,** Concentration changes of PC and PE with (3 days, CAY, purple) and without (CT, pink) CAY treatment from lipidomics (n = 3 independent experiments). **e,** Relative invasiveness of control M381 cells (normalized to 100%) and M381 after treating with 1 μM CAY, 1 μM CAY plus 5 μM OA for 3 days (n = 4 independent experiments). **f,** Two color pulse-chase experiments. Top scheme: M381 cells were firstly incubated in medium

containing 5 μM CAY for 60 h (pulse), then the medium was changed to fresh medium containing 20 μM d_{33}-OA but not CAY for 10 h (chase). Representative SRS images at the C-H lipid channel and the C-D channel were shown at two time points of 60 h (1 set, i.e. only pulsed) and 70 h (2 sets, i.e. pulse-chased representative cells are squared). **g,** Relative viability of 1 μM CAY treated M381 cells without (CT) or with rescue of 1 μM indicated unsaturated fatty acid (UFA) for 3 days (n = 4 independent experiments). PO: Palmitoleic acid; LA: Linoleic acid; ALA: Alpha-linoleic acid; EPA: Eicosapentaenoic acid; AA: Arachidonic acid. (n = 4 independent experiments). **h,** Relative viability of 5 μM CAY treated M381 cells without (CT) or with rescue of 5 μM indicated UFA for 3 days (n = 4 independent experiments). **i,** Representative ER stress marker expression with (3 days, purple) and without (pink) CAY treatment (n = 2 independent experiments). Scale bars, 20 μm. Data shown as mean ± SEM. Lipidomics data are provided as Supplementary Data 1. Source data are provided as a Source data file.

Rank	S_1 top 100	S_2 top 100	S_3 top 100	S_4 top 100	Rank	S_1 top 100	S_2 top 100	S_3 top 100	S_4 top 100
1	TYR	RXYLT1	ST8SIA5	NNMT	51	PDE6B	GLUD2	HS6ST3	TYMP
2	DCT	ALDH1A1	GALNT5	MGST1	52	HSD17B8	LPL	NPR2	RDH10
3	GYG2	CYP27A1	DHRS3	GDA	53	QDPR	CERS1	BST1	PNMT
4	GMPR	NPR1	ALDH1A3	BCAT1	54	GCNT3	GUCY2C	BHMT2	AGPAT4
5	PNPLA4	ATP6V0A4	CYB5R2	MGAT5B	55	PFKFB2	PDE7B	CSGALNACT1	PDE9A
6	QPRT	GALNT5	NT5E	HS6ST3	56	CYP19A1	ASAH1	PDE7B	ALDOC
7	RENBP	TYRP1	PLA2G7	MTAP	57	ST8SIA1	LDHD	GSTM3	PDE1C
8	ADCY2	UROC1	CHST1	GALNT14	58	GSTO2	FMO4	CYP27C1	CYP24A1
9	GALNT3	FOLH1	ACOX2	B3GALNT1	59	NAT8L	CKMT1B	PDE9A	NME7
10	GAPDHS	GAL3ST1	PDE1C	ANPEP	60	MGST1	EPHX2	AK5	MFNG
11	TYRP1	ST8SIA1	B3GNT7	PTGS2	61	RDH8	CSGALNACT1	AK6	DSE
12	PRDM7	B3GALT1	GALNT13	CHST15	62	GALNT12	TPK1	INMT	TXNRD1
13	ADCY1	TYR	B3GNT5	HS3ST3A1	63	HSD3B7	ALDH1A3	DDO	B3GNT3
14	PIP5K1B	DGKI	B3GALT2	HS3ST3B1	64	PIK3C2B	BAAT	ACSS3	PCK1
15	ALDOC	ENPP2	MGLL	CYP2S1	65	CKMT1A	PCYT1B	P4HA3	CA2
16	ATP6V0D2	ACP5	DPYD	PTGES	66	INPP4B	PDE2A	ABHD17C	NME3
17	BAAT	B3GNT7	AKR1C3	GUCY1A2	67	MAOA	SCD5	DGKA	B4GALT1
18	LPIN3	ALDH2	AKR1C1	NMNAT2	68	LFNG	ELOVL2	PLCE1	PGM2L1
19	PLB1	DCT	ST6GALNAC5	LFNG	69	ATP8	CRYL1	SYNJ2	CYP26A1
20	CA14	NT5E	TBXAS1	DPYD	70	CHAC1	B3GALT2	HSD17B3	SYNJ2
21	CHSY3	ST6GAL1	ELOVL4	ALPP	71	ALDH3B2	CKMT1A	NMRK1	PLCD3
22	PTGDS	ST8SIA5	ABAT	CYP1B1	72	PLCG2	CA12	PDE10A	ACOT4
23	LDHC	COLGALT2	MGST2	CDS1	73	NAT8	ENPP1	GFPT2	UGCG
24	TKTL1	CYP7B1	AKR1B10	GALNT6	74	NAT8B	ACOT12	COX7A1	AMPD3
25	OGDHL	ASPA	UGT8	AOX1	75	PDE11A	CYP19A1	ME1	GGT5
26	PKLR	POLR2F	PIK3CG	ASS1	76	GALM	PIK3CG	GAD1	KYNU
27	ACP5	EXTL1	HS3ST5	AKR1C3	77	ARG2	PRDM7	ACSM4	RRM2
28	CA8	RENBP	PHGDH	PLA2G16	78	GSTA1	CNDP1	OPLAH	NMNAT3
29	UGT2B7	XYLT1	LPL	B3GALT5	79	CKB	ABAT	DSE	NQO1
30	GLUL	ST3GAL6	EXTL1	ST6GAL2	80	PLD1	INPP5F	PLA2G4C	HS6ST2
31	PDE3B	MAN1C1	HEPH	GPX3	81	HSD17B6	GPD1	B3GAT1	EXT1
32	GUCY1A2	LARGE1	GPX7	MAN1A1	82	ACOT4	IL4I1	PIGZ	MBOAT7
33	CKMT1B	LARGE2	NMNAT2	XDH	83	PIP4K2A	MGAT5	HS3ST3A1	PDE6B
34	SELENOI	GAPDHS	CHST2	AK5	84	ST6GALNAC3	FADS2	AMT	PDE4D
35	ACSBG1	TBXAS1	PCYT1B	AK6	85	ISYNA1	DGKG	TUSC3	GLUL
36	GALC	PDE3B	ALDH2	INPP5J	86	ST6GALNAC1	HYAL1	FADS2	NSD1
37	PIK3CD	PDE3A	A4GALT	CPT1C	87	MAT1A	CHST11	ENPP4	NSD2
38	CYP1A1	CHST6	B4GALNT1	ENPP4	88	CYP24A1	BBOX1	UXS1	NSD3
39	ATP6V0A4	GSTM1	CHST7	CA8	89	RDH12	ACOX2	NOS2	ENTPD8
40	NPL	MAT1A	ANPEP	ACSL5	90	ACER2	GDPD1	ALDH3B1	TCIRG1
41	HNMT	MGAM	AOX1	PDE11A	91	PDE5A	ADCY2	XDH	CYP27C1
42	MGAT4A	MGAM2	FOLH1	HSD17B2	92	HYAL1	PIGZ	MFNG	HACD1
43	NMRK2	GALNT3	ETHE1	DGKE	93	HSD3B2	GLDC	IDS	PLOD2
44	B3GALT4	CA14	RXYLT1	PIK3CD	94	B3GALNT1	DDO	PLCH1	GCK
45	MTAP	ENPP3	VNN1	GBGT1	95	ASAH1	GNS	INPP5F	GSTM3
46	ST3GAL6	CHST7	PLOD2	GALC	96	MTMR8	PDXP	BCAT1	BDH1
47	FHIT	GSTA4	TPH1	PDE5A	97	LDHD	ACSL1	PGM3	ALDH1L2
48	PNLIPRP3	UGT2B7	CHST3	MOCOS	98	PDE4D	FHIT	GCNT1	ADK
49	HOGA1	HS3ST1	GALNT18	SPTLC3	99	HS6ST2	HOGA1	PTGES	IPMK
50	DGKE	MOGAT1	HS3ST1	ALPG	100	GSTO1	PNPLA4	A3GALT2	MARS

Supplementary Table 1. The genes list in Supplementary Figure 1. S_1-S_4 columns each indicates the top 100 ranked metabolic genes uniquely upregulated in the melanocytic (S_1), transitory (S_2), neural crest (S_3) and undifferentiated (S_4) phenotypes in Supplementary Figure 1.

Melanoma cell line	Phenotype	IC50 to vemurafenib (nM)	Mutational status
M262	melanocytic	150	BRAFV600E mutant<br>2 copies BRAF<br>AKT1 mutation&amp<br>CDKN2A deletion
M229	transitory	282	BRAFV600E mutant<br>4 copies BRAF<br>MITF amplification<br>AKT1 amplification<br>PTEN deletion
M397	transitory	132	BRAFV600E mutant
M409	neural-crest-like	1018	BRAFV600E mutant
M381	mesenchymal	>100000	BRAFV600E mutant

Supplementary Table 2. Basic backgrounds of the five selected melanoma cell lines used in this study.
