# Supplementary material for: Raman-guided subcellular pharmaco-metabolomics for metastatic melanoma cells
Source: Nat Commun. 2020 Sep 24;11:4830. doi: 10.1038/s41467-020-18376-x (PMC7518429; doi:10.1038/s41467-020-18376-x)
Supplement: Supplementary file 3 — Description of Additional Supplementary Files [file 41467_2020_18376_MOESM3_ESM.docx]

File Name: **Supplementary Movie 1**Description: Representative hyperspectral-SRS images stack of M381 cells across 2800-3050 cm-1 with spectral resolution of 8 cm-1

File Name: **Supplementary Data 1**

Description: Lipidomics profiling of bulk M381 cells with (CAY) and without (CT) CAY (1 μM, 3 days) treatment. n = 3 independent experiments
