# Supplementary material for: Raman-guided subcellular pharmaco-metabolomics for metastatic melanoma cells
Source: Nat Commun. 2020 Sep 24;11:4830. doi: 10.1038/s41467-020-18376-x (PMC7518429; doi:10.1038/s41467-020-18376-x)
Supplement: Supplementary file 6 — Reporting Summary [file 41467_2020_18376_MOESM6_ESM.pdf]

nature research

Corresponding author(s): Lu Wei

Last updated by author(s): Aug 04, 2020

Reporting Summary

Nature Research wishes to improve the reproducibility of the work that we publish. This form provides structure for consistency and transparency in reporting. For further information on Nature Research policies, see our Editorial Policies and the Editorial Policy Checklist.

Statistics

For all statistical analyses, confirm that the following items are present in the figure legend, table legend, main text, or Methods section.

n/a	Confirmed	
☐	☒	The exact sample size (*n*) for each experimental group/condition, given as a discrete number and unit of measurement
☐	☒	A statement on whether measurements were taken from distinct samples or whether the same sample was measured repeatedly
☐	☒	The statistical test(s) used AND whether they are one- or two-sided<br>*Only common tests should be described solely by name; describe more complex techniques in the Methods section.*
☒	☐	A description of all covariates tested
☒	☐	A description of any assumptions or corrections, such as tests of normality and adjustment for multiple comparisons
☐	☒	A full description of the statistical parameters including central tendency (e.g. means) or other basic estimates (e.g. regression coefficient) AND variation (e.g. standard deviation) or associated estimates of uncertainty (e.g. confidence intervals)
☐	☒	For null hypothesis testing, the test statistic (e.g. *F*, *t*, *r*) with confidence intervals, effect sizes, degrees of freedom and *P* value noted<br>*Give P values as exact values whenever suitable.*
☒	☐	For Bayesian analysis, information on the choice of priors and Markov chain Monte Carlo settings
☒	☐	For hierarchical and complex designs, identification of the appropriate level for tests and full reporting of outcomes
☒	☐	Estimates of effect sizes (e.g. Cohen's *d*, Pearson's *r*), indicating how they were calculated

Our web collection on statistics for biologists contains articles on many of the points above.

Software and code

Policy information about availability of computer code

Data collection	SRS images were collected with Olympus FluoView 3000. Spontaneous Raman spectra were collected with LabSpec6. RNA-seq data were mapped and aligned to human reference genome (UCSC hg 19) with TopHat. Assembled transcripts were generated from mapped reads using Cufflinks. Lipidomics data were collected by Sciex Lipidyzer platform. The authors will make all previously unreported custom computer code used in this work available upon request.
Data analysis	ImageJ/Fiji 1.0, MATLAB 2019, GSEA v4.0.1, IncuCyte Basic Analyzer v2019, Excel 12.39 and GraphPad Prism 8 were used for analysis.

For manuscripts utilizing custom algorithms or software that are central to the research but not yet described in published literature, software must be made available to editors and reviewers. We strongly encourage code deposition in a community repository (e.g. GitHub). See the Nature Research guidelines for submitting code & software for further information.

Data

Policy information about availability of data

All manuscripts must include a data availability statement. This statement should provide the following information, where applicable:

- Accession codes, unique identifiers, or web links for publicly available datasets
- A list of figures that have associated raw data
- A description of any restrictions on data availability

All the data supporting the findings of this study are available within the article and its Supplementary Information files. The source data of Figs. 1-6, and Supplementary Figs. S5-S7, S9-S13 are provided as a Source data file, and are further available from the corresponding author upon reasonable request. The following databases are used: Human Reference Genome (UCSC hg 19, http://hgdownload.cse.ucsc.edu/goldenpath/hg19/bigZips/hg19.fa.gz), Gene Expression Omnibus database (GEO, https://www.ncbi.nlm.nih.gov/geo/), Molecular Signatures Database (MSigDB, https://www.gsea-msigdb.org/gsea/msigdb/index.jsp). RNA-seq data have been deposited to array express with accession number of E-MTAB-8842. All the other data supporting the findings of this study are available

within the article and its information files and from the corresponding author upon reasonable request.

Field-specific reporting

Please select the one below that is the best fit for your research. If you are not sure, read the appropriate sections before making your selection.

[x] Life sciences [ ] Behavioural & social sciences [ ] Ecological, evolutionary & environmental sciences

For a reference copy of the document with all sections, see nature.com/documents/nr-reporting-summary-flat.pdf

Life sciences study design

All studies must disclose on these points even when the disclosure is negative.

Sample size	No sample size calculation was performed. Number of experimental was chosen based on previous experiments using similar experimental settings. For both in vitro and imaging experiments, a sample size of at least 3 was used as the minimum samples for quantification. The specific sample size is indicated in the figure legends.
Data exclusions	No data were excluded.
Replication	Experiments were performed with three different biological replicates and at least two technical replicates. All attempts at replication were successful.
Randomization	Random allocation.
Blinding	Blinding was not relevant to this study because this study did not involve clinical sample grouping etc.

Reporting for specific materials, systems and methods

We require information from authors about some types of materials, experimental systems and methods used in many studies. Here, indicate whether each material, system or method listed is relevant to your study. If you are not sure if a list item applies to your research, read the appropriate section before selecting a response.

Materials & experimental systems

n/a	Involved in the study	
x		Antibodies
	x	Eukaryotic cell lines
x		Palaeontology and archaeology
x		Animals and other organisms
x		Human research participants
x		Clinical data
x		Dual use research of concern

Methods

n/a	Involved in the study	
x		ChIP-seq
x		Flow cytometry
x		MRI-based neuroimaging

Eukaryotic cell lines

Policy information about cell lines

Cell line source(s)	M229, M262, M381, M397 and M409 melanoma cells were obtained from UCLA.
Authentication	Melanoma cell lines used in this study are patient-derived and generated under UCLA IRB approval # 11–003254.
Mycoplasma contamination	Cell lines used in this study tested negative for mycoplasma contamination
Commonly misidentified lines (See ICLAC register)	No commonly misidentified lines were used in the study.
